# Proximal Cavities

**Published:** 1863-02

**Authors:** J. S. Latimer


DENTAL REGISTER OF THE WEST.

VOL. XVII.] FEBUARY, 1863. [NO. 2.

### Original Essays and Communications.

### PROXIMAL CAVITIES.

Read before the Brooklyn Dental Association on the evening of Dec. 19, '62

BY DR. J. S. LATIMER.

*Mr. President:*—Having been honored by your appointment to prepare and read a paper before this intelligent body, upon the preparation of proximal cavities, I will proceed to state, that no proximal or other cavity is well prepared for filling until its thin and friable edges are reduced to a somewhat regular line all around its border, which is usually best effected by a judicious use of files well adapted to that delicate purpose.

Small, plain cavities do not demand the free use of the file, and hence we can not come fully at them without a separation other than that thus gained. The only method of getting at them without loss of substance, which ought to be preserved, is found in some modification of the process denominated "wedging." But, as is said, "too much of a good thing is good for nothing," I fear some are with the same mind with the Indian who said, when discussing the legitimate dose of ardent spirits, "too much is just enough !"

For want of time to prepare an entirely new paper, I offer you one the rough draft of which was written long since, but never read to any dental body on the subject of Wedges and the Mallet.

The great Yankeeism is, "I can do that," and usually has at the first opportunity the "I'll try" doctrine put in practice irrespective of lack of preparation on the part of operator, or consequences to the patient. And hence, we have abundance of examples of failures ranging all the way from the merest infinitesimal tampering long drawn out to the boldest recklessness of heroic prompt and energetic action instantly instituted, and as hastily "done! !"

I do not say that these have not their uses to fulfill to us as a body, but I do say, that they are the congeners of the hells in which fair crafts have foundered, leaving their bare poles above the smooth waters that hide their treacherous shoals that appear depths as a warning to all who may be voyaging that same way, before disintegration or malevolence had removed the testimony that just *there* had gone down as fair hull as ever danced before the lightsome breezes of inexperience and innocence!

I am about wedges like the woman when possessed of her first cooking stove, who said, I wonder *how* I *ever* got on without it, and like her find my labors wonderfully facilitated, so that a part of the time formerly consumed in the merely getting on may now be given to efficient and useful advances in principles discovered, understood and applied.

It is very evident from observation of operation, and the discussions of "filling teeth," that but very few use wedges in the process, and also of those who do use them still fewer who use them in accordance with the laws of the economy and intelligent reference to the end to be attained.

I. Very loose tender teeth are more safely, easily, and securely filled by an intelligent use of wedges and the mallet

II. Very stiff, stubborn and strong teeth are more expeditiously and perfectly filled by an intelligent use of wedges and the mallet! !

III. Very crowded and irregular, badly decayed teeth are more certainly and successfully filled by an intelligent use of wedges and the mallet ! ! !

Wedge primarily signifying "a mass," "a lump," more strictly "a mass of metal," and geometrically "a solid of five sides," with rectangular base, two rhomboidal sides meeting in an edge, and two triangular ends, "something in the form of a wedge."

*The* wedges used in dental operations, more particularly, refer to shape and density, but also include all the definitions in their various phases in the round of completeness for this especial purpose.

There are two principal results attained by wedges that is impossible without them, *i. e.* lateral support and compression of the festoon of the gum that effectually prevents bleeding so annoying when it occurs.

They are liable to do mischief in two ways dependent upon the manner of insertion and length of time they are kept "in situ." Fully to apprehend which we must bear in mind the sort of tissue that is acted upon primarily, and its peculiar circulation and quantum of life resident within it, (vis.,) periosteum with colorless circulation (blood).

A certain amount of pressure, applied in a given time, extravassates this fluid which must either absorb or organize, the first of which never does harm, the latter of which always in a degree compromises the well being of the organ involved.

The first and more common way that mischief is done, by an unjudicious use of the wedge is excitement, of periosteal irritation and inflammation.

The second is more rare, but not less destructive to the well-being of the tooth ; it occurs in cases of fangs that are not straight, where the wedge so binds the apex of fang against the side of the socket (alveolus), as to arrest the circulation to the pulp at the foramen ; if this be kept up for any considerable length of time, mischief is imminent, although in good constitutions even this is not always certain death to the pulp. (This statement will suggest to the observant mind not to move the tooth too far out of its normal line, which is best secured by the use of wedges between the spaces of one or two adjoining teeth each way from the one operated upon, thereby distributing the force to all in the arch as far as touching naturally or by intervention of these distributors of force, the wedges so inserted.)*

Operators of reputation and in full practice are very liable to use the wedge too long before excavating and filling; some from want of time, others from want of attention to the diversity and necessity of the cases, insert rubber wedges, and send the patient away with the indefinite "call again" instruction. (Let me say it here, *never* insert a *rubber* wedge if you are not able to see it, two or three times a day, for we know not what an hour may bring forth—and if you fail to take it in the "nick of time" you must either postpone or excite troublesome irritation that may *never* leave the tooth while living!!)

To be successful we must operate before irritation is set up or await its subsidence. And hence, the wedging and filling should be done at the same sitting, thus exciting no irritation of periosteum in the great majority of the cases who are likely to apply for this sort of service.

---

*Many teeth are completely anæsthicized by the compression of the circulation by the wedges, so that we may excavate them with impunity.

All are benefited in this respect by the benumbing influence of the pressure, so that we are more than compensated for the unpleasantness of the hasty separation by this prompt means of even tender teeth, and irritable gums; to say nothing of the immunity gained from periosteal irritations and inflammations so very common when the slow and so called "easy" method of separating with rubber wedges is pursued. Then the time we save to patient and operator should not be regarded as a small item in the account. This arises from the possibility of proceeding uninterruptedly in preparing, filling, and finishing, all three of which processes will be accomplished in less time than the excavation alone where the patient cringes at each cut of the excavator so as to embarrass the process of the work in any hands sympathetic enough to fit them to perform the work with a tenderness commensurate with the highest success.

It is simply dishonest, wicked, to insert a wedge and send the patient away for the sake of gaining *time*, or retaining the case by making them think it necessary, when we know it is not. Unfortunate for them and us, if we *do think* it necessary, as the trial will prove to all who give the requisite attention to know the sequel.

The very best organizations only are capable of passing such an ordeal without serious mischief. A useful and efficient mode of applying rubber wedges is in the shape of rings cut from tubing and slipped over the crowns of the teeth to be operated on, so as to embrace their necks tightly, effectually compressing the festoons of gum ; if applied at the same sitting, becomes a source of danger if allowed to act over night, or through any considerable length of time, by gradually but surely extracting the teeth by the leverage of gum and process between which and the crown the resistance of the rubber insinuates itself.

Material for wedges, and form into which wrought will vary in the different classes of teeth as well as in the variety of constitutions—the molars, other things being equal, requiring thicker and harder than the bicuspids and single-fanged teeth in front of arch. Shape should conform to the space where they are to be used. Sharp corners should be avoided. Gradual taper facilitates the insertion.

Metals, wood, bone, horn, fibrous substances and gums have their separate claims in the cases to which adapted. Hard resistant teeth must be met by a like hardness and persistance ; soft, loose teeth by the gums and softer woods ; teeth of intermediate character requiring intermediate materials and modes.

Fibrous wedges serve a good purpose in this range, such as twines of cotton, linen, silk, etc., etc.

Tortuous cavities do not admit of their margins being sustained by wooden, bone, or horn wedges, and metal ones would have to be so carved as to render them impractical. So to sum up the constant use of them will soon enable the versatile mind to readily elect what sort to prescribe in a given time. Reckless force and indiscriminate haste make no part of superior workmanship with the wedge, the mallet or the ordinary force of hand, arm and body.

An equal amount of discretion, earnestness and skill, and a little practice, will, I am confident, pronounce for safety, facility, beauty and permanence in favor of using wedges and the mallet.

